# Ice edge failure process and modelling ice pressure

**DOI:** 10.1098/rsta.2017.0340

**Published:** 2018-08-20

**Authors:** Kaj Riska

**Affiliations:** Offshore Engineering Section, Faculty of Civil Engineering and Geosciences, Delft University of Technology, 2628 CN Delft, The Netherlands

**Keywords:** ice–structure contact, ice pressure, ice failure, ice strength, ice crushing process

## Abstract

Ice action on ships and offshore structures is commonly determined by calculating the contact ice pressure. The aim of this paper is to describe the empirical background for determining the ice pressure. This review article describes six different test series where ice edge indentation and contact ice pressure have been investigated. These test series are ice pressure measurements onboard IB Sisu in the Baltic in 1977, pendulum tests carried out at Arctec in Ottawa, Canada, in 1979, laboratory and full scale ice crushing tests at WARC in 1988 and onboard IB Sampo 1989, medium scale indentation tests on Hobson's Choice Ice Island 1990, ice crushing tests at NRC, Ottawa 1992 and the JOIA tests in Hokkaido 1996–1999. These tests were selected as at each series a new phenomenon was observed. The aim of the paper is to introduce the main features for ice–structure contact empirically through the description of tests. The paper is concluded with a short description of the existing models for ice pressure, especially to gain an insight and highlight the main observations in each test series and how the models for ice pressure have developed based on the observations.

This article is part of the theme issue ‘Modelling of sea-ice phenomena’.

## 1. Introduction

When a ship collides with an ice edge or ice cover drifts against a stationary structure, the force acting between the structure and ice is transmitted through the contact between the structure and ice. As the contact (loosely) includes an area where the contact exists, instead of the contact force, the contact is described by contact pressure, as this quantity is easier than contact force to relate with ice characteristics (see e.g. [1]). While the motions and deformations of the ice feature and the structure have a general influence on the contact, the local ice failure—or rather the local ice strength—has the largest influence on the contact pressure. Thus ice pressure can be considered as a ‘local’ phenomenon. In the literature of ice action, the term ‘local’ is used with many meanings, usually in contrast with ‘global’. Here ‘local’ refers to ice deformation and a failure occurring at the contact. One immediate note in this description is that ice is always considered to break—in most cases ice is forced to fail to let the ship progress further or the ice cover drift to continue. In some cases, the relative motion stops as the force required to break the ice is large enough to stop the relative motion either by stopping the motion altogether or by ice and the structure starting to move together.

The present description of ice edge failure and ice pressure reviews a set of experiments carried out to observe the contact between an indenter (in full scale structure/ship) and ice edge, and also to measure the ice pressure. The paper does not present any new results but tries to put measurements already made in the context of understanding ice pressure. The selection of the measurements described is naturally very subjective, and a quick apology is offered to those who feel that their measurements have been neglected. The paper is completed by a short review of the models suggested for describing or determining the ice pressure.

Many of the figures are reproduced from the original sources as no other figures or references are available. Thus the quality of these leaves a bit to be desired.

## 2. Background

Ice pressure is defined as the pressure acting on the interface between the structure and ice edge. This definition immediately awakens two main questions. Firstly, why ‘ice edge’? When a level ice sheet drifts against a stationary structure that offers a vertical side against the ice, the meaning of ice edge and its geometry is clear. When a ship proceeds in level ice, the geometry of the edge is less clear. The ice edge arises from the repeated ice failure in bending, which leaves the ship to collide repeatedly on the edge of the newly broken ice.

The second question is related to the ‘interface’. It sounds clear that there is a *direct contact* between ice and the outer shell of the structure. The fact that ice often fails by crushing, i.e. being broken into a granular material containing also very small ice particles, obscures the idea of a direct contact that is defined to occur between intact ice and the structure. There may be a direct contact in the interface and also contact through the crushed ice.

Local ice edge cracking can also create a contact that is smaller than what is usually described as the *nominal contact area*. The nominal contact area is the area where the contact would be if the geometries of the structure and the ice feature were extended to the contact without considering deformation or especially cracking. Practically, all the models for ice pressure use the concept of the nominal contact area to define the ice pressure; see, for example, the collection of models in [2]. It should be noted in passing that most formulations use the so-called *projected nominal area*. This is the nominal area projected on a tangent plane of the contact that is normal to the relative velocity.

**Figure 1.**
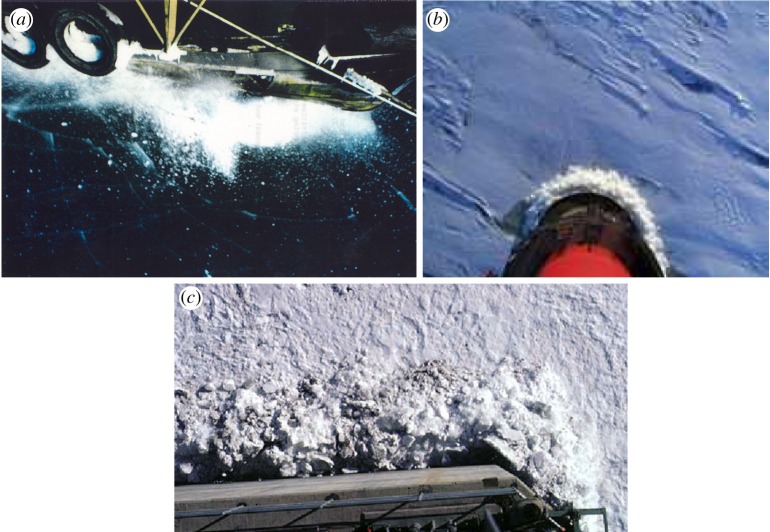
(*a*) The crushed ice in front of an ice-breaking ship showing also the set of radial and circumferential cracks. The level ice thickness is about 20 cm. The ship is proceeding towards the bottom right (photo: Antti Joensuu). (*b*) Crushed ice in front of the lighthouse Nordströmsgrund [3]. (*c*) Crushed ice in front of a vertical flat surface (photo Devinder Sodhi). (Online version in colour.)

When a structure indents an ice edge, the ice edge is described to fail in ‘crushing’. Figure 1 shows the crushing of an ice edge when a ship is progressing in ice and the ice failure when ice cover drifts against a vertical pile or wall. Both cases show extrusion of apparently finely broken ice. Visual observation of the piles of crushed ice in front of stationary structures shows that the piece size distribution is wide with quite large pieces (meaning ice pieces the maximum dimension of which is of the order of the level of ice thickness); see, for example, figure 1. In the case of a vertical column, the nominal contact area can be easily determined; it is *A*_nom_ *=* *π*/2*·D·h_i_*, where *D* is pile (column) diameter and *h_i_* ice thickness. The projected nominal area is simply *A*_proj_ = *D·h_i_.* In the ship case, the contact areas are much more difficult to determine as the edge geometry is not clear—obscured by the crushed ice even in more clear cases as in figure 1. The importance of the distinction between the different areas becomes clear when perusing the results from different ice pressure measurement campaigns.

**Figure 2.**
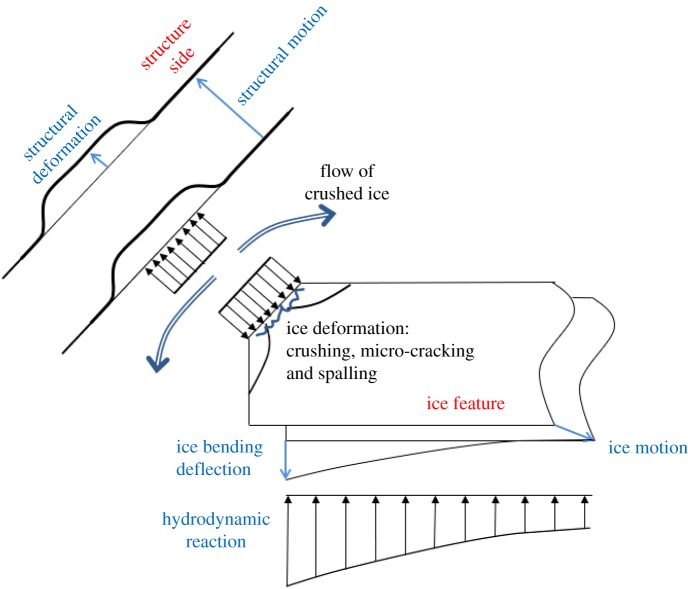
A sketch of the deformations and motions of the structure and ice in the structure–ice interaction. (Online version in colour.)

In the modelling of structure–ice interaction, all the deformation and motion components should be taken into account especially to determine the relative motion between ice and structure. Figure 2 shows a sketch of the components that are to be taken into account in modelling the structure–ice or ship–ice interaction. In order to focus the description of measurements that follows, some observations from this sketch (and the state of assumptions made at present) can be made (for a discussion on these, see [4]):

(a) ice pressure is assumed to be uniform over the whole nominal contact area and also on the projected nominal contact area;
(b) the local structural response can be elastic or plastic;
(c) global structural deformation is (linear) elastic;
(d) local ice failure is either described as ‘crushing’ or spalling by cracks created at the contact;
(e) the hydrodynamic reaction force has a static part (due to buoyancy) and a dynamic part (due to accelerating water); studies like Keijdener *et al.* [5] show that, at least in ship cases, the dynamic part cannot be neglected;
(f) the length of the contact (in a direction normal to the plane of the figure) is taken into account only in very simple geometries; and
(g) ice bending is modelled as a simple plate on an elastic foundation.

Figure 2 is drawn having a ship–level ice contact as background. A similar figure could be drawn for vertical structures; ice bending and the hydrodynamic reaction would not be present then. Here the spalling or flaking as it has also been called [6] is shown in two dimensions. The flakes formed by cracks are, however, three-dimensional as the crack surface curves up towards the contact. The size of flakes formed has an influence too.

The measurements carried out have especially focused on the following items:

1. effect of temperature and indentation speed on the magnitude of ice pressure and failure process;
2. the ratio of the actual contact area, i.e. area where the pressure is not zero, to the nominal contact area; and
3. ice pressure distribution and magnitude on both the nominal and actual contact area.

## 3. Contact ice pressure measurement campaigns

Ice pressure has been and is measured by measuring the deformation caused by ice pressure on a selected area. This may be the deformation of the structural outer shell or a purpose-built panel within the structural shell. In any case, the pressure is obtained based on the force–deformation characteristics of the gauge, i.e. pressure is obtained from the measured force, which is obtained from the measured deformation. Numerical or physical calibration to obtain the force–deformation relationship is done using a certain gauge area. This area in many cases is clear especially if the gauge consists of a separate face against ice. The gauge calibration usually assumes a uniform pressure on the gauge area, an assumption that is more exact the smaller the gauge area is.

Mechanical gauges cannot be used to get an idea of the pressure distribution as the gauges cannot cover the whole surfaces without gaps. Two electric gauges have been used that can be used to improve the situation: polyvinylidene fluoride (PVDF) film, which is piezo-electric [7], and the so-called tactile sensors; see, for example, Sodhi [8]. These two methods have their disadvantages, for example, in calibration, but offer a method to cover almost the whole instrumented surface without gaps and be used to consequently gain an understanding of the actual contact area and pressure distribution on it.

In the following, six measurement campaigns are described in which ice pressure was measured and the ice edge failure observed. The presentation follows chronological order. The ice used in these tests has a large influence on ice pressure value. Thus the ice used is described to the extent it is possible based on the original sources. The grain size or direction is not described in most of the tests—only the test series in \S3b and \S3e report it. It is unfortunately not possible to describe all similar campaigns and the selection here serves an aim to develop further the modelling of ice pressure—this is described as the last section of the paper.

### (a) Ice pressure measurements onboard icebreaker Sisu

**Figure 3.**
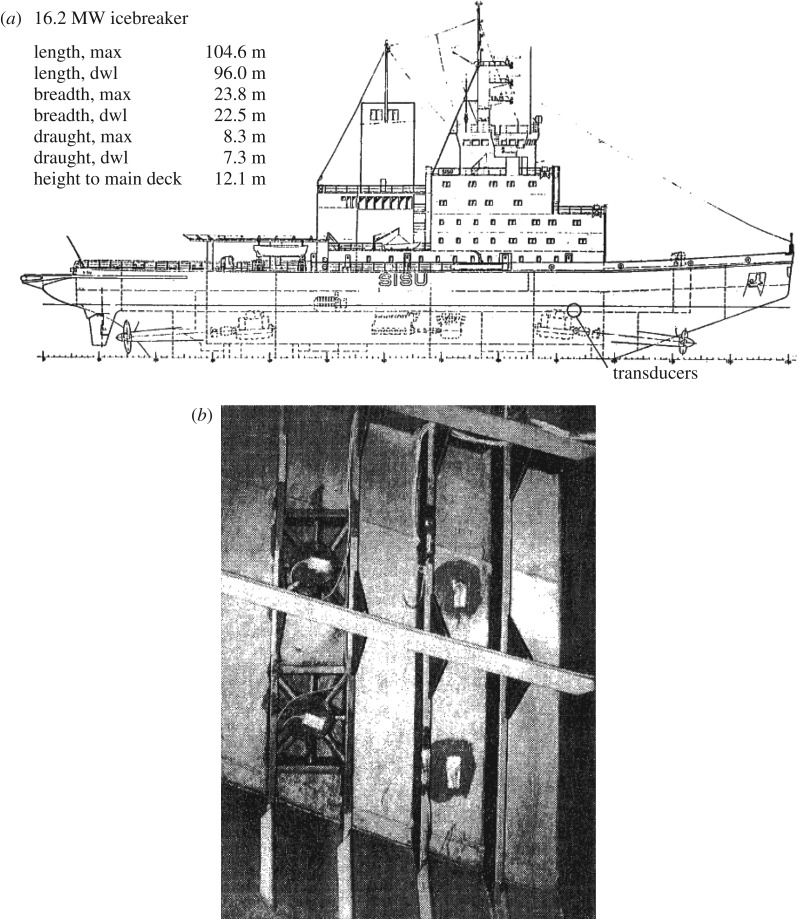
(*a*) Icebreaker Sisu and the location of the gauges [9]. (*b*) A photograph of the pressure gauges (left) and the strain gauges on the shell plating (right) [9]. The photograph is taken normal to ship plating, the four vertical frames in the photo are vertical while the one longitudinal frame (slightly tilted in the photo) is actually horizontal.

The Finnish icebreaker Sisu was instrumented with pressure gauges for winter 1978; see Vuorio *et al.* [9]. The pressure gauges consist of a cylinder (inner diameter 20 cm) that was rigidly attached to the shell of the icebreaker (thickness 32 mm, *σ_Y_* = 235 MPa). The deflection of the icebreaker plating inside the cylinder perimeter was measured relative to the cylinder. The calibration with a uniform pressure yields the deflection versus pressure. The gauges were calibrated using the finite element (FE) method and also with an external hydrostatic pressure. Besides the pressure sensors, gauges to measure the stress on the shell plating in the longitudinal direction (normal to frames) were installed (figure 3). These ‘stress gauges' are based on a special arrangement of strain gauges.

During the measurements, the icebreaker operated the whole winter in the northern Baltic. The maximum level of ice thickness varied during the measurements from 45 cm at the beginning of January, to a maximum of about 85 cm in early April and finally decreased to about 75 cm at the beginning of May. The icebreaker operation consisted of escorting ships to and from northern ports. The usual escort speeds can be up to about 12 knots.

**Figure 4.**
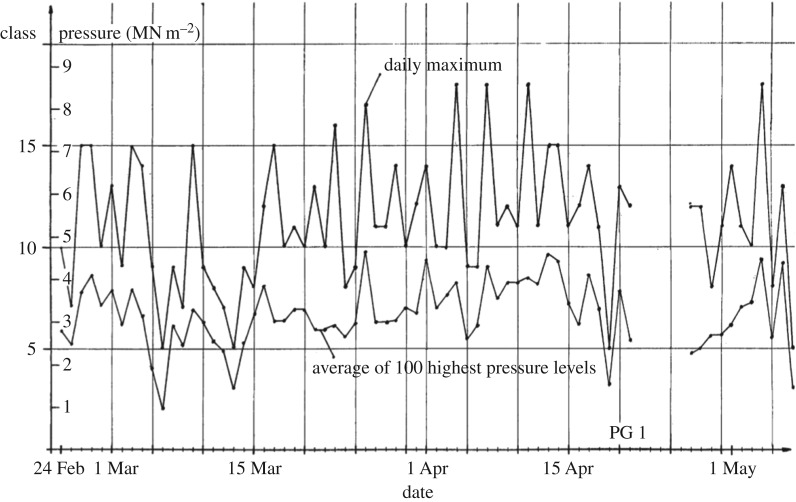
The measured daily maximum ice pressures [9].

The ice pressure was not recorded as time histories but rather as a collection of pressure peaks. The measured daily maxima of ice pressure are shown in figure 4. The pressure readings were converted into 32 classes (bins). As the figure shows, the maximum measured pressure was about 8.5 MPa.

**Figure 5.**
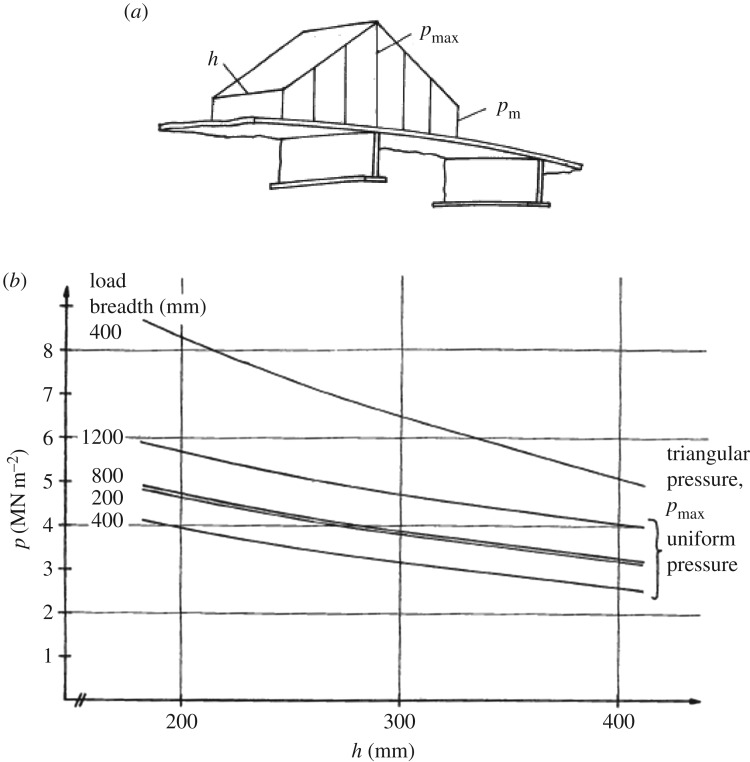
(*a*) A part of the assumed pressure distribution. Load height is *h*. If the load length is more than one frame spacing, the triangular shape continues in both directions [9]. (*b*) The maximum pressure *p* versus load height *h* at different load patch dimensions which give the maximum stress measured in plating, 170 MPa, based on FE calculations [9].

The stress in the plating was measured in the adjacent frame spacing to the pressure gauges. The measured stresses did not tally with the measured pressure if the pressure is assumed to be uniform. Thus a hypothesis stemming from the theory of stiffened plating resting on an elastic foundation was made, i.e. that the stiffer part of the shell carries more pressure. This was simplified to a triangular pressure distribution as shown in figure 5*a*. Thereafter, FE calculations were made using different pressure patch dimensions (length and height *h*), to find the maximum pressure *p*_max_ value (the value of the uniform pressure) that gives the maximum stress measured. This maximum stress was 70% of the yield, i.e. about 170 MPa. The results of calculations are shown in figure 5*b* as the maximum pressure versus load patch height using the load patch length as a parameter. As the measured maximum pressure is about 8.5 MPa, acting as an assumption on a stiff area, it can be seen that a relatively small load height (about 20 cm) and a triangular shape fit the data best. It should be noted here that the minimum pressure *p*_m_ was kept at 2.4 MPa, which was assumed to be the uniaxial horizontal compressive strength of Baltic ice.

Conclusions from the pressure measurements onboard IB Sisu:

— the measured ice pressure is clearly higher than the uniaxial compressive strength of ice (which is about 4 MPa), up to 8.5 MPa;
— the ice pressure distribution is not uniform; and
— high pressures are measured in spring (beginning of May) when ice is melting; this is intriguing as the strength of melting ice is very low (see, for example, [10]).

All these conclusions were surprising at the time of these measurements but were put into some physical context later, using more detailed measurements (as described below).

### (b) Pendulum tests at Arctec Inc. in Ottawa

**Figure 6.**
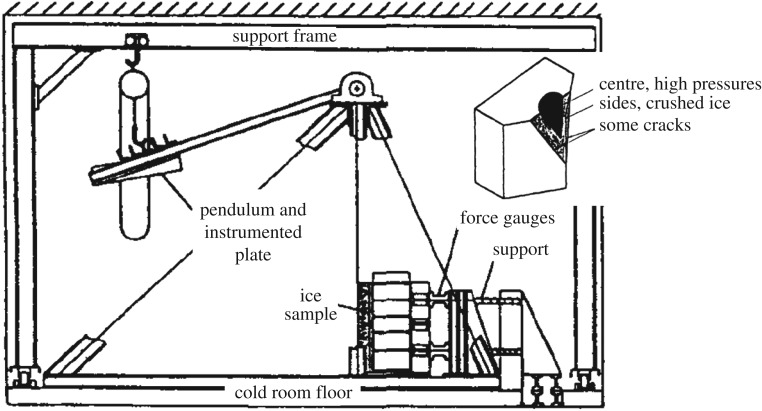
The set-up in the pendulum tests and the ice sample after a test (inset) (modified from [11]).

The high ice pressures measured with the Sisu instrumentation stimulated a laboratory test series with high indentation speeds. This resulted in a test series where the high speeds, up to about 5.6 m s^−1^, were achieved using a pendulum by Glen & Comfort [11] (figure 6). The tests were carried out in the cold room of Arctec Inc., in Ottawa, Canada, in 1981. Ice used in the tests was sea ice with relatively low salinity, between 1 and 6 ppt. Ice temperature was also varied, being between −2 and −25°C. The ice samples were wedge shaped and the top corner of the wedge was impacted upon. Total force and local ice pressures were measured. The pressure sensors had a circular active area with a diameter of about 1 inch (2.5 cm). The pressure sensor array was relatively sparse, with distances between sensors of about 5 cm.

**Figure 7.**
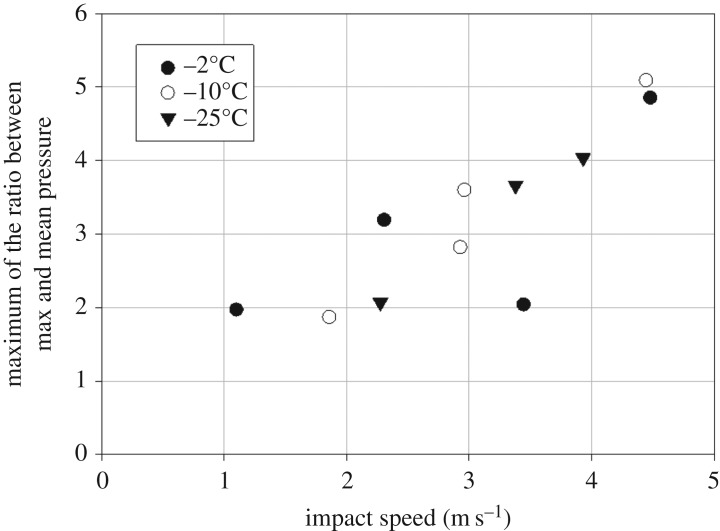
The maximum ratio of the measured maximum and mean ice pressure versus impact speed (modified from [11]).

The measured maximum local pressure was about 38 MPa. The impact speed was noticed to influence the ice pressure so that the maximum pressure peaks became in relative terms larger; the ice temperature, on the other hand, did not have a large influence, as figure 7 shows.

**Figure 8.**
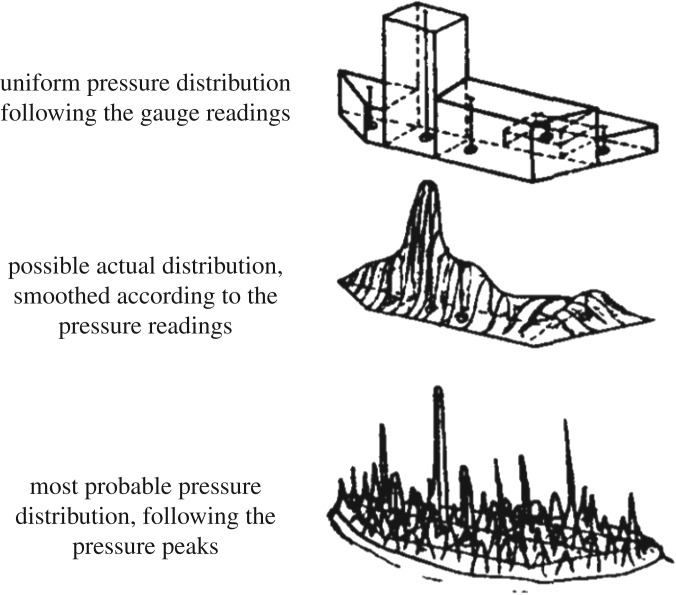
Possible ice pressure distributions on the nominal contact area according to Glen & Comfort [11]. These overestimate the measured normal force by a factor of 3, 2 or 1.5, respectively, from the top.

The very high pressures—much higher than in the Sisu measurements—given by the local pressure measurements were surprising, as the measured pressure was an order of magnitude larger than the uniaxial compressive strength. The researchers tried to match the measured pressures and an assumed pressure distribution to the measured total force. This was done by assuming some pressure distribution that matches the pressure measured by pressure gauges. The area on which this is done is the nominal contact area. The first figure is a uniform pressure on an area allocated for each pressure gauge, the second is a smoothed distribution and the third one is a peak distribution. Even a peaked pressure distribution overestimated the force by a factor of 1.5 (figure 8).

This measurement series was the first one that observed very high local ice pressures. In the context of the Korzhavin [1] formulation, the high pressures were attributed to a three-dimensional stress field having a confining effect, but the ratios of uniaxial to multiaxial strength stated this way were only up to about three. The high very local pressures measured are explained by an open-ended macroscopic ice failure surface along the hydrostatic pressure axis, with the limiting mechanism provided by a phase change under pressure as explained by Gagnon [12]. Pressure melting occurs at a pressure of about 100 MPa at −10°C; see, for example, Hobbs [13]. About macroscopic failure surfaces for ice, see, for example, Riska & Frederking [14].

### (c) Laboratory indentation tests at WARC, Helsinki

A large set of ice crushing experiments was carried out in 1988 at the Wärtsilä Arctic Research Centre (WARC) with the cooperation of the Helsinki University of Technology (at present Aalto University). The intent was to measure the ice pressure with PVDF film (see [7]) and at the same time observe the ice–indenter contact through a transparent, Lexan, plate. PVDF is a piezo-electric material sensitive to the pressure applied to it. The tests have been reported in laboratory reports [15,16] and one general paper [17]. The description that follows is based mainly on the second report.

**Figure 9.**
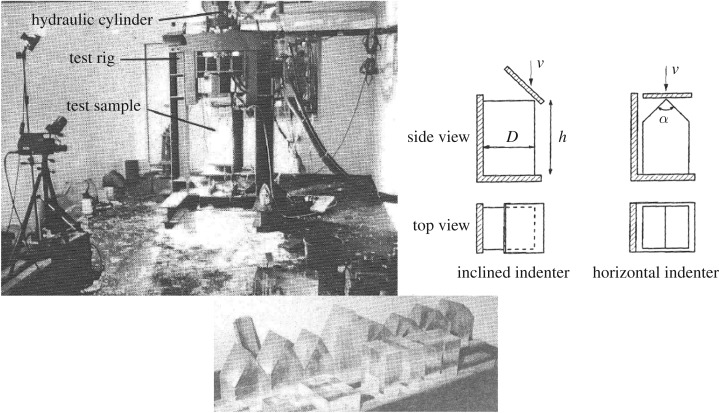
A view from the test room, the two different crushing test types and a view of prepared test specimens [15].

The test specimen cross section was always 25 × 25 cm (height 65 cm) while the edge that was crushed was either the edge of a rectangle with an inclined indenting plate or a wedge (figure 9). The tests were carried out with brackish ice from the northern Baltic. The temperature was kept during the tests at about −6°C. The indentation speeds were 5, 10 and 15 cm s^−1^. The indenter plate was either covered by the PVDF pressure sensors or was transparent, enabling filming through the plate. Apart from the ice pressure, the normal and tangential force were measured and naturally the indentation.

**Figure 10.**
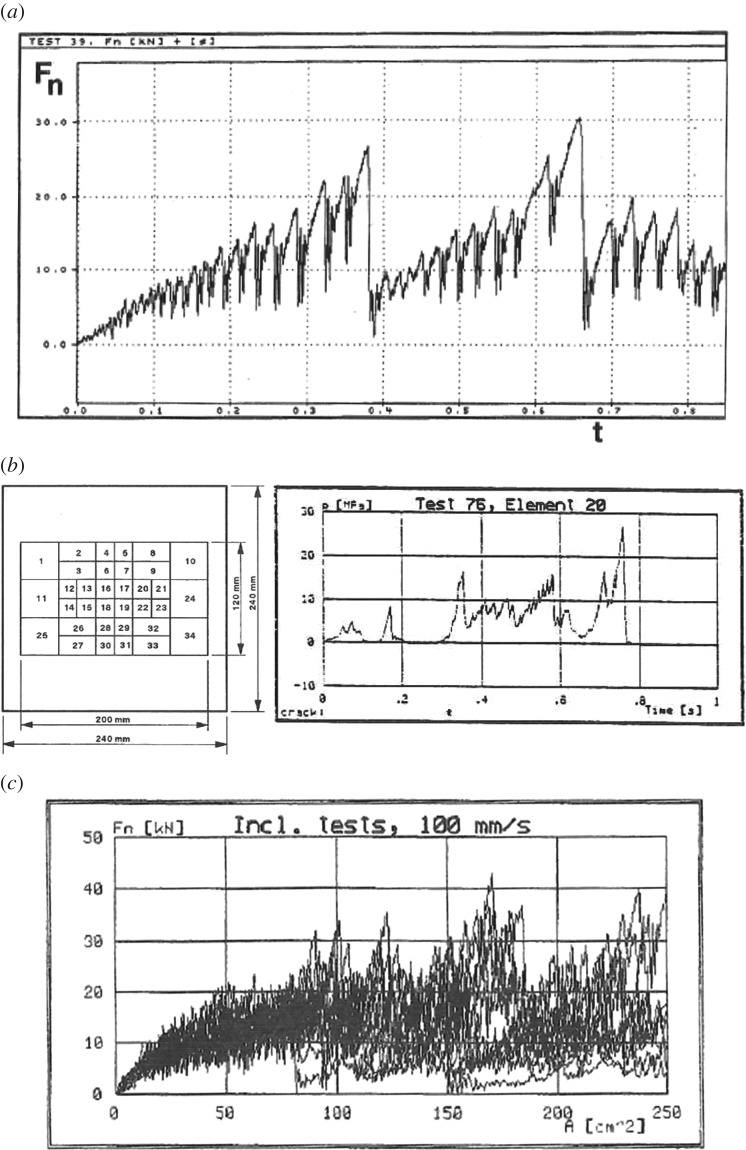
(*a*) A time history of the normal force from a test with wedge shaped specimen [15]. (*b*) The geometry of the PVDF ice pressure plate and one example of measured ice pressures on the smallest size of PVDF elements, about 1.5 × 1.5 cm [15]. (*c*) A collection of normal force–time histories from indentation tests on an ice edge at 10 cm s^−1^ indentation speed. *A* is the nominal area deduced from the displacement and the test specimen geometry and *F*_n_ is the normal force on the contact area [16].

A typical normal force–time history is shown in figure 10. It consists of triangular peaks that grow in size when the indentation increases. At some point, a larger failure in the sample occurs and the process starts again. The local ice pressure did not follow the triangular pattern as figure 10 shows. The largest pressure measured in the laboratory tests was about 36 MPa. The force measurements suggested a form of the edge indentation force. A collection of force–time histories from edge indentation tests is collected in figure 10*c*. If the focus is on the beginning of the test, before the first major crack (thus up to an area of about 50 cm^2^), it is clear that the trend is not linear with the increasing area but rather somewhat below the linear. If a power fit is done to the first part, i.e. a fit as *F*_n_*(t)* *=* *CA(t)^*m*^*, then the area exponent is between −0.28 and −0.43. This is referred to as a process pressure–area relationship—mainly to distinguish it from a pressure–area relationship where pressure peaks from different tests are plotted (see, for example, the figure at the end of this subsection).

**Figure 11.**
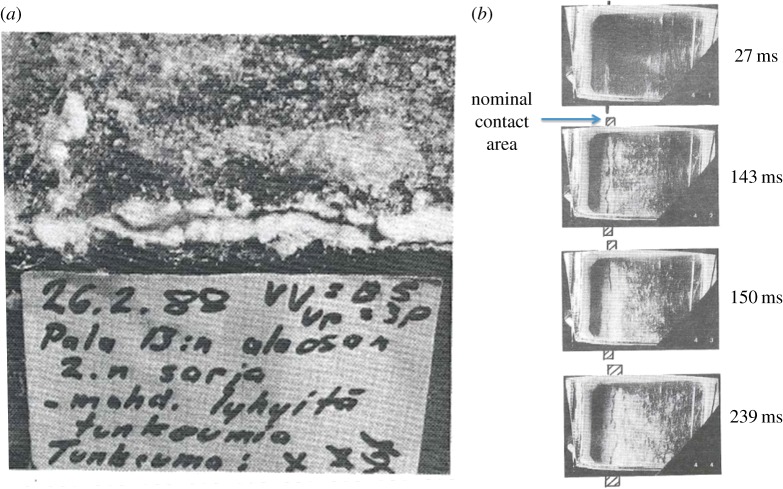
The edge of a rectangular specimen after an indentation of about 1 cm (*a*) and photographs through the indenter [15]. (Online version in colour.)

The measured pressures were not, however, the main new feature in these indentation tests. This came from the visual observations of the contact area. The contact seems to be transmitted through a small narrow band that is shown black in photographs—black, as the external light disappears in ice at the direct contact. Figure 11 illustrates this finding. The PVDF elements, even if they were somewhat large for this observation, endorse this observation that a linear high-pressure area is formed. The correlation between pressure sensors was almost zero in any direction from any chosen element except with the adjacent elements where the correlation was slightly below 0.5.

**Figure 12.**
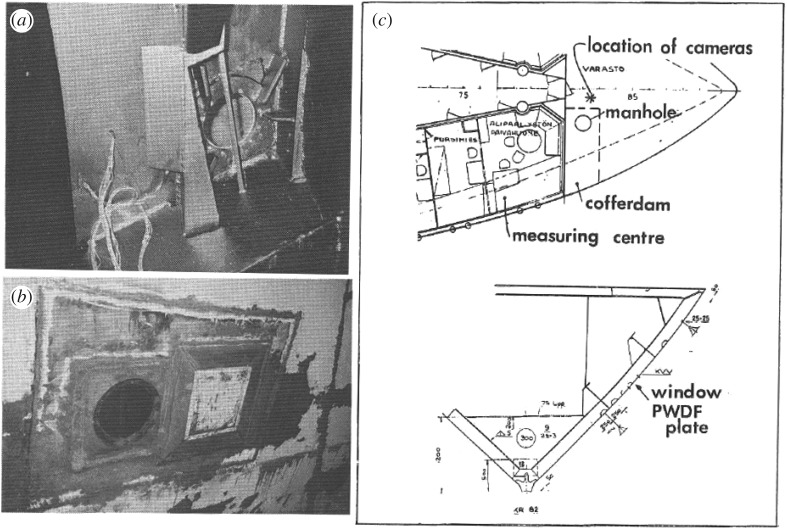
An internal (*a*) and external (*b*) photo of the plate and window and the location of the PVDF plate and the window (*c*) [16].

**Figure 13.**
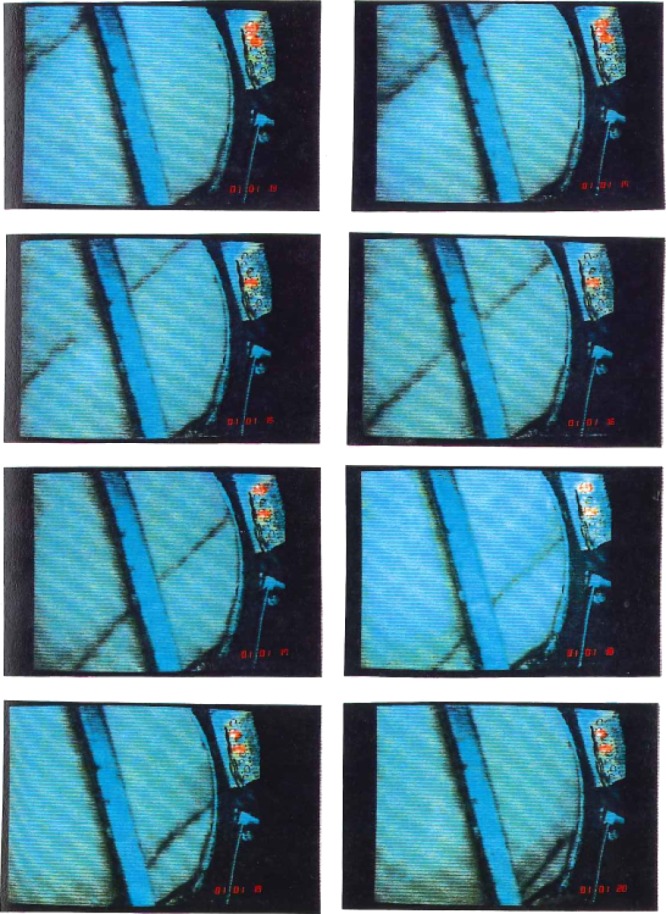
A view through the window on the icebreaker. The time proceeds from left to right and down. The almost vertical line in the middle of the window is a bracket supporting the window [16].

The observation of the narrow high-pressure band induced the question whether this is an artefact from laboratory tests. A test in full scale was decided to be done onboard icebreaker Sampo. IB Sampo is an old Finnish line icebreaker that now acts as a tourist cruise icebreaker at the port of the northern city Kemi. The PVDF plate was attached to the hull and beside the PVDF plate a window was made at the waterline of the icebreaker (figure 12). The tests were carried out outside Kemi port in February 1989. The pressure measurements gave similar results as in the laboratory, including the narrow high-pressure band feature. An example of the visual observations is shown in figure 13.

In figure 13, the narrow band observed in the window is horizontal, even if the location of the camera relative to the window gives an inclined line. The line moves down as the icebreaker is breaking the level ice in bending and the ice floe is pushed down. The total time between the first frame and the last frame is about 100 ms.

**Figure 14.**
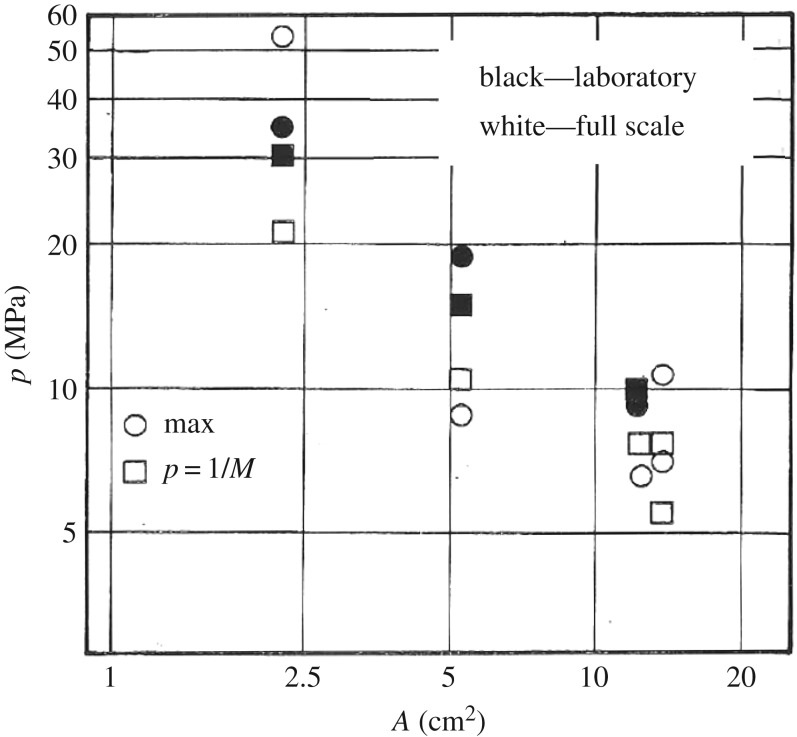
The maximum measured pressure on each PVDF element size plotted versus the active area from laboratory and ice breaker tests. The points labelled as 1/*M* are based on statistical fitting on the pressure histogram where *M* is the number of tests included [16].

One final observation from these laboratory and full scale indentation tests can be made. When plotting the maximum pressure measured in the tests on the different size of pressure gauges, a decreasing trend versus gauge area is noticed (figure 14). If a fit of a power relationship (*p* *=* *CA*^*n*^) is made (similar to figure 10*c*), an area exponent of about −0.5 is obtained. This is somewhat steeper than the process pressure–area relationship—having an area exponent of about −0.3 to −0.4—fitted on the collection of force–time histories. It is, however, important that both the process pressure–area relationship (figure 10*c*) and the one on maxima (figure 14) follow the same decreasing trend versus area.

### (d) Medium scale indentation tests, 1990

**Figure 15.**
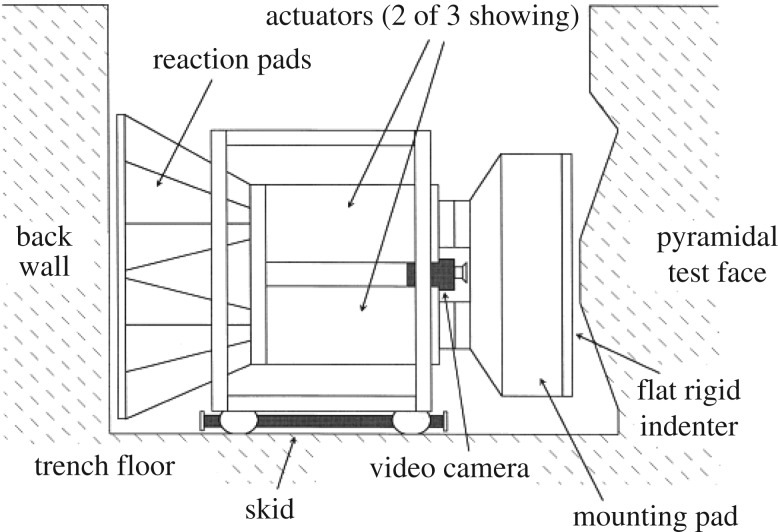
The test set-up in the medium scale indentation tests [19].

**Figure 16.**
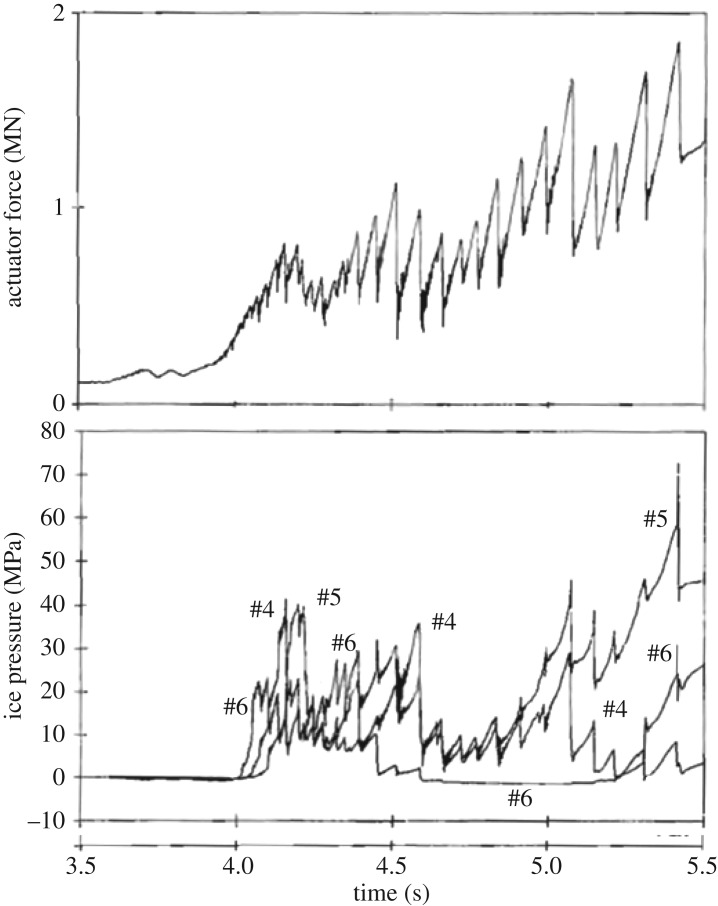
Measured contact force and some local ice pressures from test #6 (modified from [18]). Indentation rate 19 mm s^−1^.

Indentation tests were carried out on an ice island named Hobson's Choice in 1990 just north of the Canadian Arctic archipelago. The tests are reported in Frederking *et al.* [18] and for example in Gagnon [12]. The ice island consists of multi-year ice and is about 45 m thick. The tests were carried out by excavating a large trench of cross section 3 × 3 m in the ice, placing a hydraulic actuator in the trench supported by the other side of the trench wall, forming a conical shaped (pyramidal) edge on the other side and finally crushing the cone (figure 15). Indentation force was measured and also two types of pressure gauges were used (PVDF and traditional ones). Forces and pressures measured in the test #6 are shown in figure 16. The similar triangular pattern as in the tests at WARC is seen in the total force–time history. The highest local pressure was about 75 MPa on the 13 mm diameter pressure gauges, which fits rather well with the pressures in figure 10*c*—surprisingly as the ice used in these two test series was different. It is noteworthy that the measured maximum average pressure was 22 MPa, and that these maximum average pressures when plotted versus nominal contact area gave rather low area exponents, about −1. This value of the exponent actually suggests a constant force.

**Figure 17.**
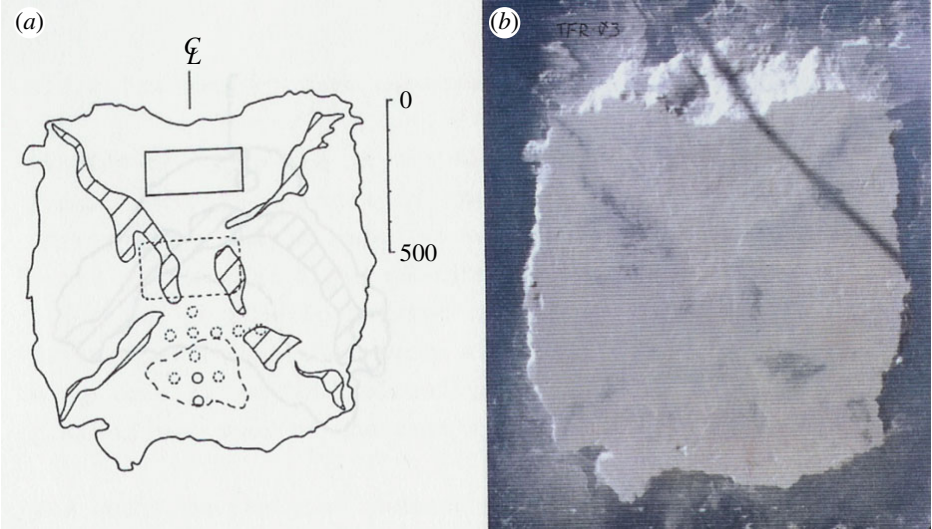
A photograph of the final contact area after a test (*b*) and the interpretation of the high-pressure areas (*a*) [20]. (Online version in colour.)

**Figure 18.**
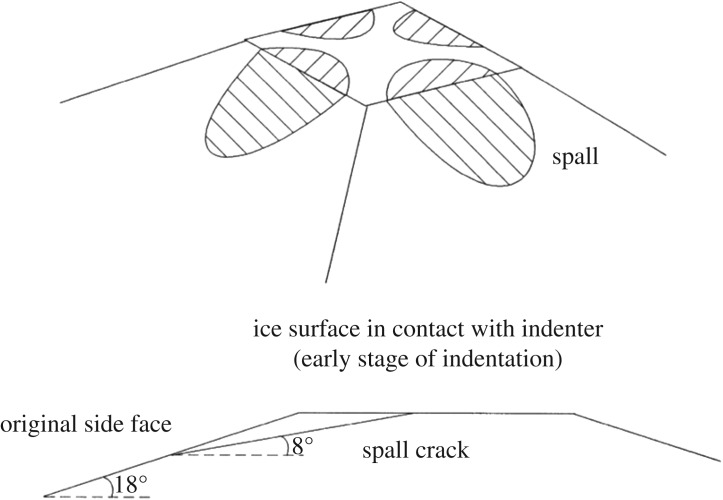
The formation of the high-pressure areas based on spall cracks [19].

An interesting observation in these tests was that even if the cones (pyramids) were quite flat—angle about 18°—linear high-pressure areas were detected. These are shown in figure 17 from test #3. It has been observed that the linear high-pressure area is usually oriented towards corners of the nominal contact area. Gagnon [12] and Gagnon & Bugden [21] have investigated this and offer an explanation based on the formation of cracks that create spalls, as figure 18 suggests. The spalling crack creates flakes that are three-dimensional. The three-dimensionality can be investigated by calculating the correlation between adjacent pressure gauges; this correlation gives an idea of the size of the flakes along the spall cracks, and their role in creating the linear high-pressure area was first suggested but in two dimensions based on the indentation tests in Helsinki (see [15]) by Daley [6]. An explanation of the narrow high-pressure band based on fracture mechanics was offered by Dempsey *et al.* [22].

### (e) Ice crushing tests at NRC, Ottawa, 1992

**Figure 19.**
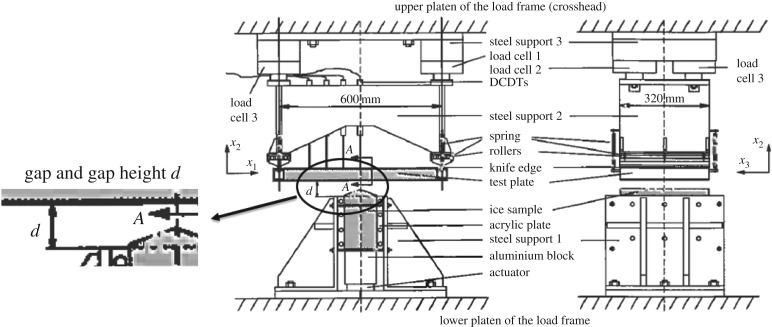
The test set-up in the indentation tests at NRC (modified from [23]). Note that the ice specimen is pushed up.

An ice edge indentation test series was carried out at the National Research Council of Canada (NRC) cold rooms at the Montreal Road campus in Ottawa in 1992. The aim of the tests was to investigate in detail the effect on the ice crushing process—ice edge indentation—of several factors, like the grain orientation of S2 (columnar grained) ice, the extrusion of the crushed ice and flexibility of the indenter. The tests are reported extensively in Tuhkuri [23–25]). The test set-up is shown in figure 19. The test specimens of size 120 × 320 × 200 mm were pushed against a test plate and forces were measured. The indentation speeds were up to 50 mm s^−1^. Ice was fresh water ice and kept in all tests at a temperature of −10°C.

**Figure 20.**
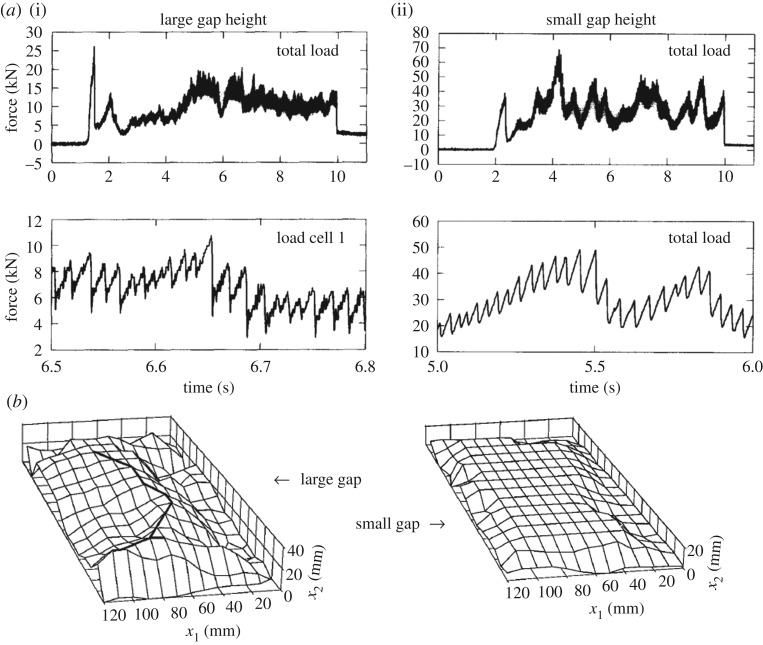
(*a*) Measured normal force in two tests, with large gap height (i) and small gap height (ii) [23]. (*b*) A sketch of the ice surface after the test [23].

**Figure 21.**
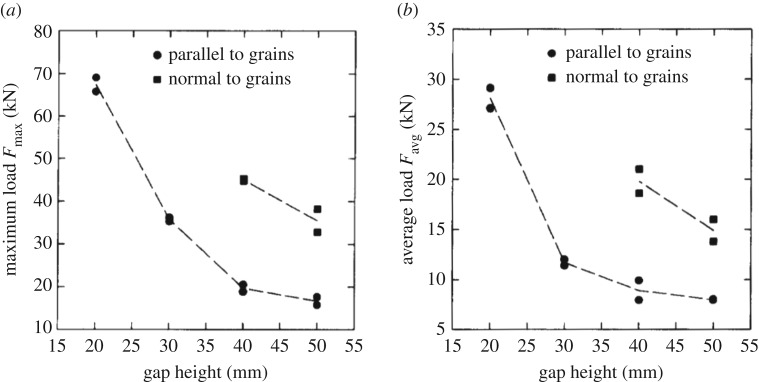
The effect of gap height and grain direction (parallel and normal refer to the long axis of columnar grains versus the indenter plate). In determining the average and maximum force, the first peak was omitted as this was deemed to be due to the impact, not the indentation process itself [24].

The measured time history shows much variation during the test but, looking a bit more closely, it is noticed that the time history consists of a triangular pattern (figure 20). The dominating frequency in the large gap case is much larger, 65 Hz, than in the small gap case, 24 Hz. Also, the actual contact area changes with gap height, as seen in figure 20. The gap height influenced the force magnitude a great deal (figure 21). The small gap height makes the extrusion of broken ice more difficult, and at the same time, the confinement increased due to the side support but also presumably due to increased pressure in the broken ice extruded from the contact. The tests made it clear that the flow of crushed ice from the contact zone has a large influence on the ice edge indentation process.

**Figure 22.**
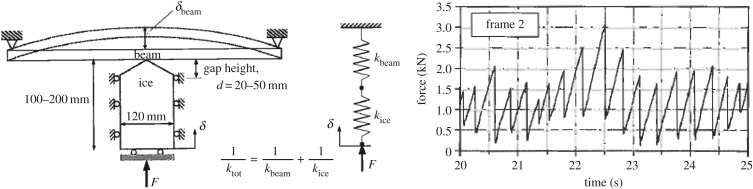
The test using a flexible plate and the resulting force–time history [23].

Tests were carried out with a flexible plate (figure 22). In this figure also the flexibility of the plate and ice (based on its elastic constants) are noted. It was noted that the rate of change of the triangular pulses (for the ascending part of the time history) in the force–time history corresponds to the total stiffness of the system as
3.1
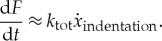

This suggests that the ice elasticity must be taken into account when modelling the ice–structure contact.

**Figure 23.**
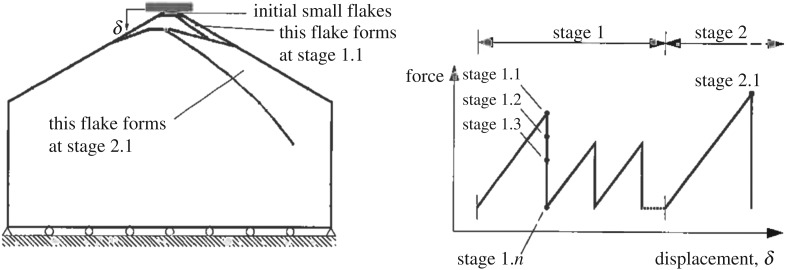
Calculated crack paths and a corresponding sketch for the force–time history [25].

Finally, it should be mentioned that the NRC indentation tests were used to investigate the spalling cracks that were observed. The study is reported in Tuhkuri [25]. The cracking was modelled by the boundary element method and several possible initial crack positions were studied. It was expected that the calculated crack paths would turn towards the wedge surface but this was not the case; the cracks run towards the interior of the sample and mostly towards the vertical side (figure 23). Naturally it is probable that, after a quite vertical crack, the next crack would run towards the former crack surface. A fracture mechanics-based explanation for the linear high-pressure zone was given, but an ice edge indentation process model is still to be developed.

### (f) Medium scale indentation test in Hokkaido

**Figure 24.**
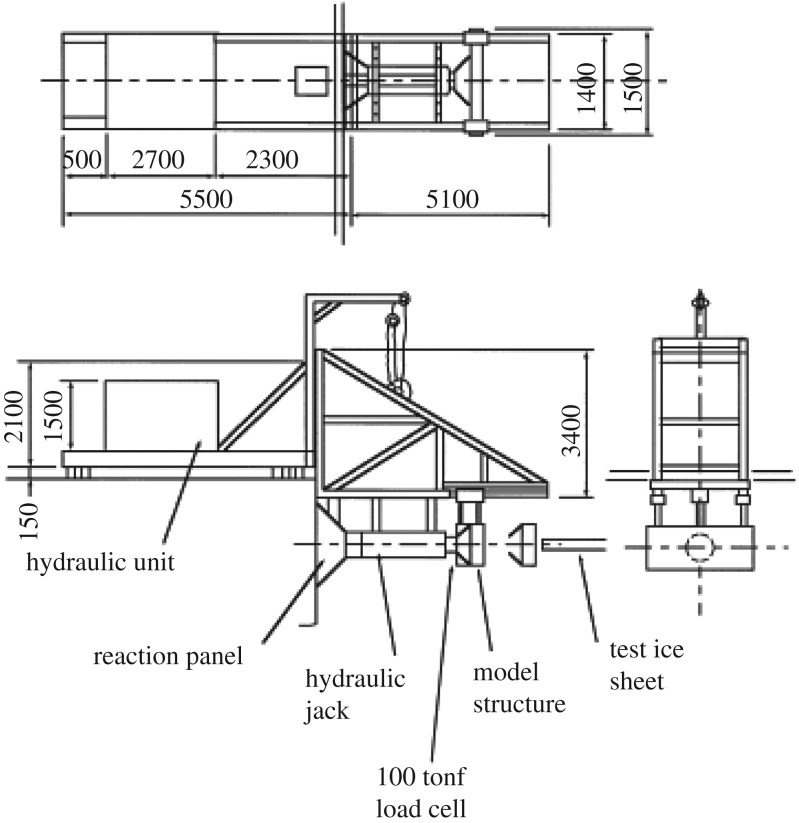
The test set-up in the medium scale indentation tests in Hokkaido. The hydraulic unit is on the quay, and the reaction panel is also restrained by the quay. The test ice sheet is floating and thus it is restrained by the surrounding ice sheet [28].

Medium scale indentation tests were carried out in Hokkaido over several years. The tests that are referred to here were carried out in winter 1998 and reported in Sodhi *et al.* [26]—earlier tests with the same apparatus were carried out for example in 1996; see Takeuchi *et al.* [27]. The test series are sometimes collectively referred to as JOIA tests to note the sponsor, Japan Ocean Industries Association. The tests were carried out *in situ* on sea ice with salinity of about 5 ppt. The test ice was floating and an apparatus was installed to indent the ice edge (figure 24). Air temperature during the tests was about −3°C. Ice thicknesses used were between 24 and 45 cm with a width of the indenter of 1.5 m. Three indentation speeds were used: 0.3, 3 and 30 mm s^−1^. Apart from the total force, ice pressures were measured with tactile sensors which enabled a measurement of the pressure distribution on the whole nominal contact area—this was a major step forward in studying ice–structure contact. The tactile sensors consisted of a plastic sheet encasing a transducer panel; there were conducting surfaces along horizontal and vertical directions in a grid pattern with a spacing of 5.4 mm. There were 44 rows and 44 columns of grid points in each panel. With the help of software developed by the manufacturer of the pressure sensing panel, the pressure was measured at each grid point, which had an area of 5.4 × 5.4 mm [26]. More details about these pressure sensors are given for example in Izumiyama *et al.* [29].

**Figure 25.**
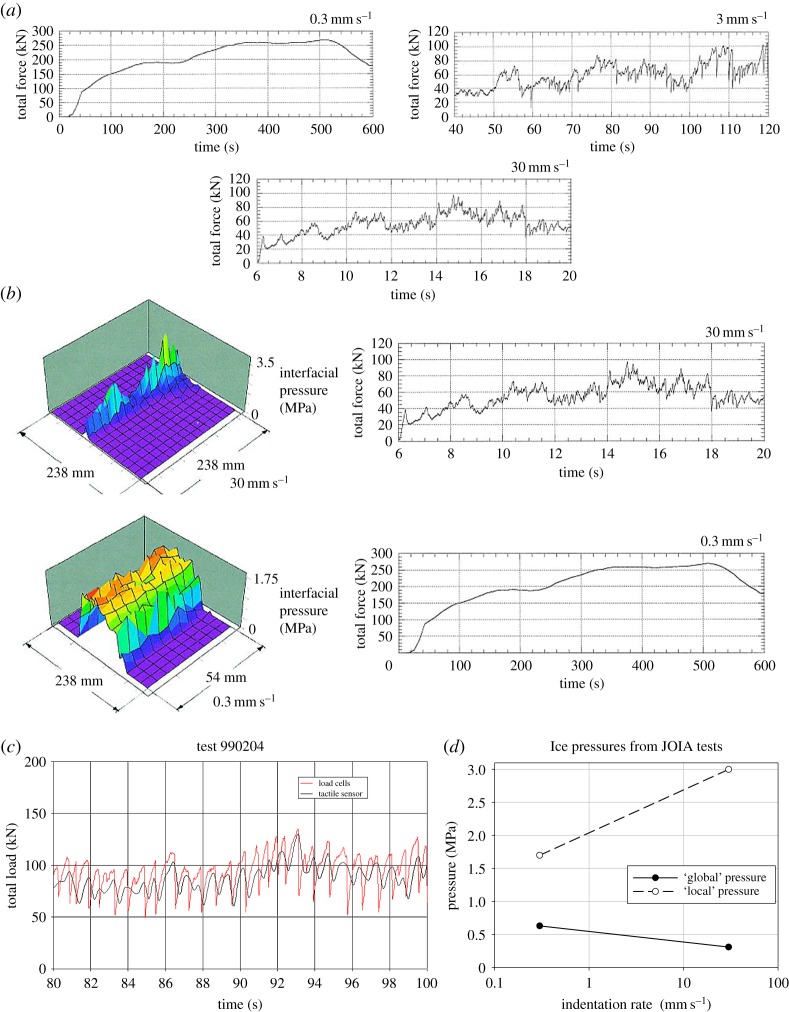
(*a*) The indentation force–time history from tests with three different indentation speeds and presumably the same thickness, 29 cm (the reference Sodhi *et al.* [26] does not very well identify each test) [26]. (*b*) The measured ice pressure distribution in two tests together with the indentation force–time history (ice thickness 29 cm) [26]. (*c*) A close-up of the force–time history from an indentation test reported in Sodhi *et al.* [28], obtained as described by Sodhi (D. Sodhi 2018, personal communication). (*d*) Maximum local and global ice pressure from tests with low and high indentation speeds. Here ‘local’ refers to the tactile sensor measurements and ‘global’ to the average pressure.

Time histories of the indentation force at different indentation speeds are shown in figure 25. There are two noteworthy features: one is that the triangular pattern is missing in some tests and the other is the large effect of the indentation speed. The triangular pattern in the force–time history was not, however, totally absent as a close-up of a force–time history shows (figure 25*c*). This triangular pattern (D. Sodhi 2018, personal communication) relates to colder ice temperature; unfortunately, no exact temperature value is available. Sodhi *et al.* [26] describe the failure with the two higher indentation speeds as ‘brittle flaking’ in figure 25*a* while the lowest indentation speed failure is ‘ductile’. This is interesting as ‘brittle flaking’ is often coupled with triangular force peaks.

It is also noteworthy that the pressures measured with the tactile sensors (small active area) are much higher with higher indentation speeds than with lower ones. This should be contrasted with the fact that the average (global?) ice pressure on the nominal area is higher at the lower indentation speeds. This is demonstrated in figure 25*d*. The observation on local and global pressures induces the question on describing the pressure versus the nominal contact area: Should the indentation speed be included as a parameter?

**Figure 26.**
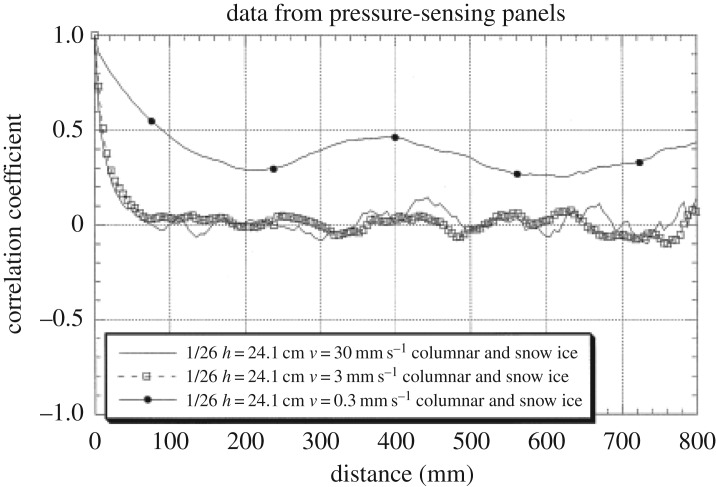
The spatial correlation between tactile pressure sensors versus the distance between sensors. The tests were carried out on 26 January 1998 with an ice thickness of 24 cm [26].

The observations made from the tests described the ice failure at the high indentation speeds as brittle flaking. This flaking created a narrow linear high-pressure area (band) where the individual pressure peaks look like a mountain range. The visual observation by Joensuu & Riska [15] shows that the linear feature looks continuous. A spatial correlation calculation from the tests in Hokkaido shows that the continuity is a visual effect, as the correlation disappears at about 7 cm distance from any location (figure 26). The spatial correlation is influenced by the failure mode, especially the size of the three-dimensional flakes forming in the brittle flaking process. The question is if the correlation is also dependent on the elastic deformation of ice as some models on ice-induced vibrations claim; see, for example, Hendrikse [30].

### (g) Summary of the measurements

Results from several test series about ice–structure interaction through ice edge indentation were described above. Some immediate conclusions from the tests can be made, such as the following:

— There seems to be a mode change in ice failure from ductile (viscous/plastic) at low indentation speeds to brittle (cracking, spall formation) at high indentation speeds. JOIA tests with relatively warm sea ice suggest that the mode change occurs between the indentation rates of 0.3 mm s^−1^ and 3 mm s^−1^.
— At higher indentation speeds the high pressure is concentrated on a narrow band that aligns with the nominal contact area geometry, preferably along the long geometric axis of the area.
— Contact area with ice is much larger in the ductile regime than in the brittle regime. Local ice pressures are, however, higher in the brittle regime whereas average pressure is higher in the ductile regime.
— The flow of crushed ice has an influence on contact; with the restricted flow the contact area becomes larger.
— In the ductile regime, the contact pressure is (more) uniform whereas in the brittle regime, the contact pressure consists of peaks that are not much correlated. This gives validity to the suggestion that the ice failure is by spalling in the brittle regime and that the spalls formed are essentially three-dimensional.
— The triangular pattern was present in most tests where the force–time history could be measured. The reason for this is allocated to a failure mode change from ductile (low indentation speeds) to brittle (higher indentation speeds). Only in some of the tests in the last test series described (JOIA), the triangular pattern was missing from some tests even if ice failure was described as brittle flaking.

## 4. Models of ice-induced pressure

It may be of interest to look at the various models for ice pressure that have been suggested. It is important to keep the above conclusions in mind when investigating the different ice pressure models. In the following, a brief review of models presented for ice pressure is made.

### (a) Models based on ice compressive strength

The first model for ice pressure assumed pressure to act uniformly on the nominal contact area. If tangential (frictional) forces are ignored, then the pressure is uniform also on the projected nominal contact area. The magnitude of pressure was assumed to be proportional to ice compressive strength. The resulting equation is called the Korzhavin equation as it originated from Korzhavin [1] based on observations made mostly on bridge piers that were vertical. Observations suggested that the proportionality factors include a shape effect based on the cross sectional shape (*m*, value 0.9 to 1), indentation factor based on the multi-dimensional stress state in ice (*I*, value 1 to 3) and contact factor based on the non-simultaneity of the contacts within the nominal contact area (*c*, value 0.3–1). Thus the Korzhavin ice pressure is
4.1
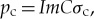

where *σ*_c_ is the compressive strength of ice.

The Korzhavin equation has been much criticised, as the coefficients are somewhat arbitrary; see, for example, Palmer & Croasdale [31]. The other drawback is that the compressive strength of ice is actually not a material constant (see, for example, Kendall [32]), but depends on the test specimen preparation and testing apparatus. The Korzhavin equation has also been applied mostly on offshore structures; the assumption of proportionality between ice pressure and compressive strength has been also used as an indication for ice pressure on ships.

**Figure 27.**
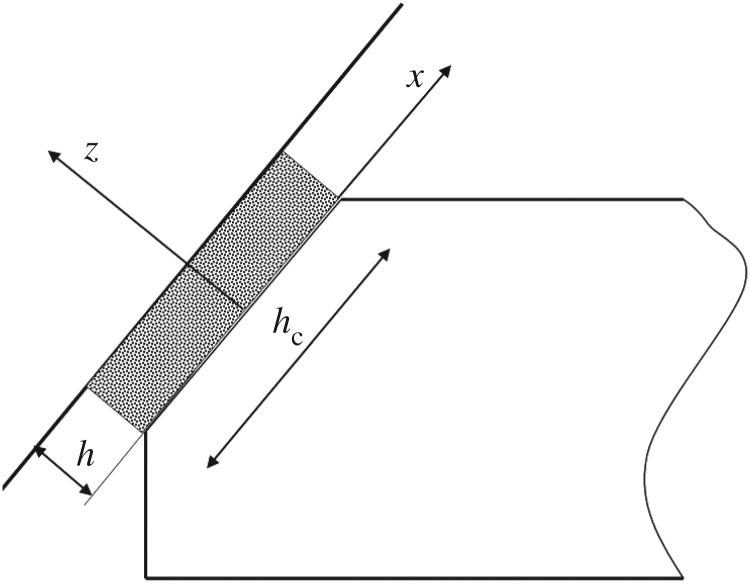
The layer of crushed ice between intact ice and the structure. The figure represents a vertical cross section normal to the contact with load patch height *h*_c_ and the crushed ice layer thickness *h*.

A formulation that was developed in view of ship ice loads is based on the assumption of a crushed ice layer between the structure and intact ice; see, for example Kurdjumov & Kheisin [33]. Figure 27 illustrates the crushed ice layer. This model is based on squeezing the crushed ice away from the contact zone. It is called generally the ‘hydrodynamic’ model for ice pressure. The pressure in the layer is derived based on Reynolds thin-film equations assuming a constant thickness of the layer and that the crushed ice behaves as a viscous fluid. The resulting pressure distribution along the contact height is parabolic (symmetrical versus the mid-point), and zero at the contact edges (this formulation is modified from [34]):
4.2
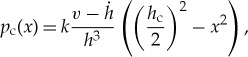

where *v* is the indentation speed and *k* an empirical parameter representing both the strength of ice and the viscosity of the crushed ice. The pressure magnitude was obtained using the so-called specific crushing energy that was determined mainly by drop ball tests where the energy consumed to crush a certain volume of ice was calculated. This pressure model was used in developing a theory for ship ice loads by Popov *et al.* [34]. Russian ship ice class rules are based on this theory.

Similar criticism as to the Korzhavin equation can be directed to the hydrodynamic ice pressure model, i.e. the description that ice strength is empirical and based on compressive strength. Further, the value of viscosity of crushed ice—and in general the assumption of viscous fluid—is somewhat problematic; see, for example, Sayed & Frederking [35]. Further, a uniform ice failure process would result in a non-uniform film thickness due to the increased mass flow towards the edges of the contact.

**Figure 28.**
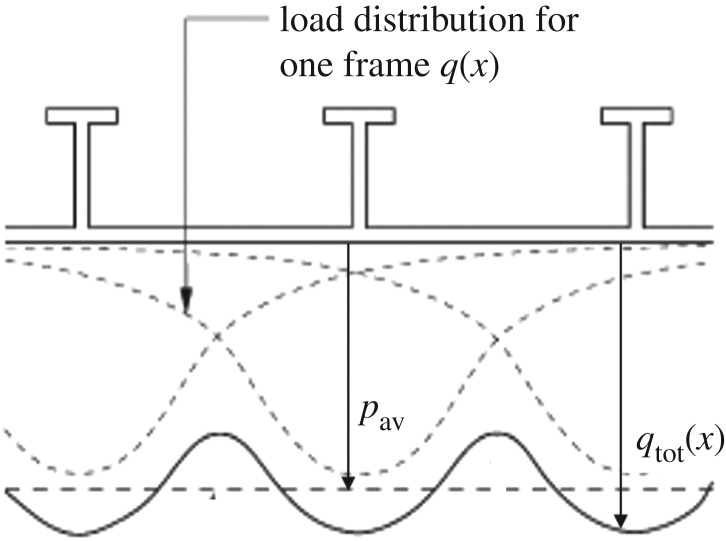
The pressure shape on stiffened ship plating resting on a Winkler foundation [36]. *x* is the coordinate along the plate.

The hydrodynamic ice pressure theory does not state anything about the pressure distribution in horizontal (longitudinal) direction. The measurements onboard IB Sisu have been used to develop the theory of the pressure distribution in the longitudinal direction for transversely framed ships. This is based on a stiffened plate resting on a Winkler foundation; see Riska *et al.* [36]. The crushed ice can be modelled as a Winkler foundation which is characterized by elasticity but does not support any shear forces—thus if the deformation of the foundation is *w*, the pressure required to create this is *p* *=* *kw*, where *k* is the foundation modulus. The situation modelled is shown in figure 28.

The shape of the ice pressure is given as
4.3
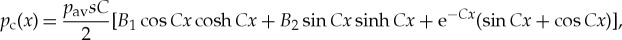

where the constants are (*k* is the foundation Winkler coefficient, *E* steel Young's modulus, *ν* steel Poisson's ratio, *t* plate thickness and *s* frame spacing)

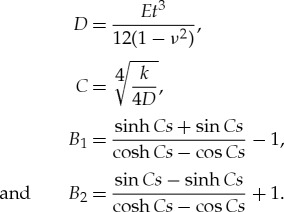

The average pressure *p*_av_ must be determined by some other means, usually empirically, as for the other formulations above.

The criticisms presented towards the Winkler foundation model are mostly focused on the foundation model; if the contact is mediated by crushed ice, the foundation model should be different, and if by direct contact, then the foundation model should include contact with elastic solid. The pressure shape has been, however, validated with two test cases; see Riska *et al.* [36].

### (b) Ice pressure dependence on nominal contact area

**Figure 29.**
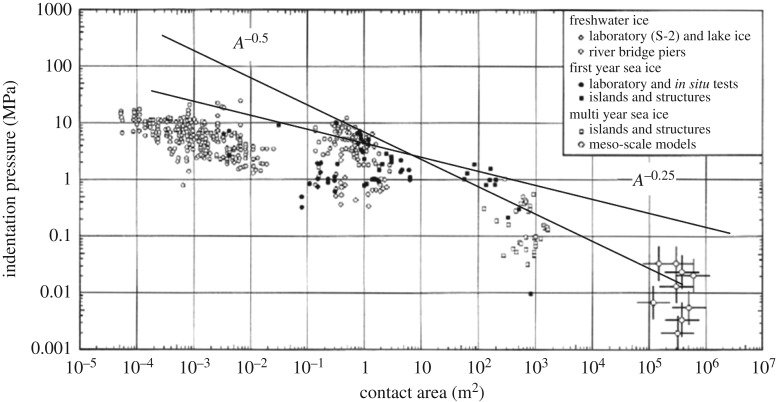
The pressure–area curve presented by Sanderson [37], modified from the original source to show the lines for pressure being proportional to *A*^−0.5^ and *A*^−0.25^.

Sanderson [37] collected measured peak ice pressure values and plotted them versus the area (figure 29). The origin for this curve is not clear but one of the earliest suggestions for it is based on ice pressure measurements onboard CANMAR Kigoriak in 1981; see CANMAR [38] and the original measurement report by Riska *et al.* [39]. The areas used in the pressure–area plots are mostly the gauge areas, i.e. based on the sensors used. This relationship has been investigated and discussed much; see, for example, Masterson & Frederking [40] and Timco & Sudom [41].

The pressure–area relationship is often called a scale effect as it introduces a geometric scale for ice pressure. As such, the measurements used to derive it do not suggest any pressure distribution on the area and the pressure used in the plots is simply the average pressure on the sensor active area—usually the sensor calibration assumes a uniform pressure distribution. Figure 29 is extended to very large area, but figure 14 shows that the pressure drop versus the area persists also in very small areas. Actually, it has been suggested that the large areas in figure 29 include different ice failure mode when compared to the areas below, say, 10 m^2^.

**Figure 30.**
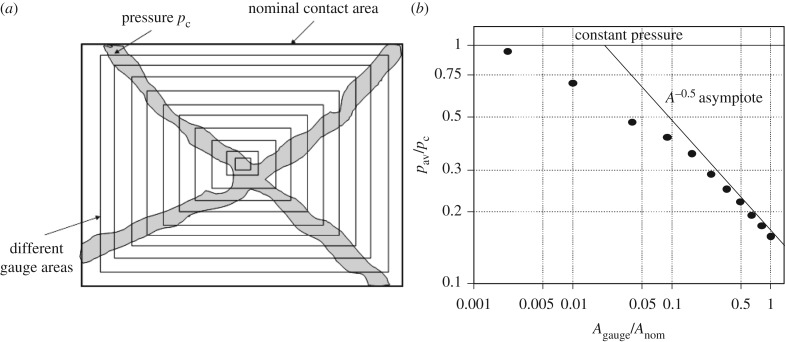
The assumed area when the ice pressure is *p*_c_ and marked with grey (*a*) and the resulting normalized measurement points plotted versus the normalized area [42].

A simple thought experiment shows that the pressure–area relationship is connected with brittle ice failure probably by spalling if it is assumed that the pressure along the direct contact in the test result shown in figure 17 is *p*_c_ and zero on other areas, and that this pressure is measured with sensors having different rectangular active area as shown in figure 30. The resulting plot of normalized ice pressures versus the normalized area follows in larger areas the pressure–area relationship with an area exponent of −0.5. If brittle fracture by spalling is required for the formation of the narrow high-pressure band, then in the ductile region (higher temperatures or slow indentation rates) the pressure–area relationship may change. It should be noted that many points used to deduce the pressure–area relationship are from ship tests where the indentation rates are high. An indirect indication of the failure mode change versus indentation speed, and the corresponding effect on pressures, is obtained from studies of ice-induced vibrations of slender vertical structures like bridge piers; the vibrations that have been observed do not occur in numerical simulations if the mode change is not introduced in the models; see, for example, Hendrikse [30]. The basis for the narrow high-pressure band has also been investigated using fracture mechanics; see, for example, Dempsey *et al.* [22].

The application of the pressure–area relationship is not straightforward in design. In principle the local structures, especially steel structures, should be designed so that different areas are used and the response checked with these. The design area (load patch) is the one that gives the largest response. This principle is followed, for example, in the Finnish–Swedish Ice Class Rules [43] where the basic ice action quantity is the line load *q* which essentially represents the averaged pressure over the contact height (*q* *=* *p*_c_·*h*_c_). The pressure–area relationship is converted in this case to a normalized line load versus load length (*l_a_*) relationship, which for the rules is assumed to be of form (for background, see [44])
4.4
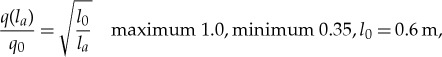

where the normalizing factor *q_0_* depends on the ice class. The application of this relationship would require checking many load lengths but the rules define the length giving the (approximately) largest response for each structural element. Offshore regulations, even if using the pressure–area relationships, do not recognize this question of the design load patch being dependent on the particular pressure–area relationship used; see ISO [2].

## 5. Conclusion

The description of several ice edge indentation test series with ice pressure measurements has shown the gradual increase of insight into the complexities of describing and modelling ice pressure. The ice failure mode is influenced by the indentation rate (and ice temperature) so that at high indentation rates (or low temperatures) ice failure is brittle. The failure mode influences the pressure magnitude, thus different indentation speed regimes (ductile, brittle) give presumably different pressures. This indentation speed effect on pressure magnitude is stated to come from ice compressive strength dependence on stress or strain rate; see Michel & Toussaint [45] and Peyton [46]. These tests suggest that ice strength increases versus strain rate up to a peak around a strain rate of 10^−3^ s^−1^ and decreases then to settle on a constant value at very high strain rates.

Controlled indentation tests in high indentation rates or compressive strength tests at high strain rates are very difficult to conduct. Results from special measurement techniques (Hopkinson split bar) have suggested that the drop in ice strength that is assumed to take place at higher strain rates does not occur; see for example, Shazly *et al.* [47] and Ortiz *et al.* [48].

Much ice pressure data come from measurements with ships where the indentation rates are presumably high. These data are, however, somewhat ambiguous, as the indentation rates are high but temperatures are close to melting point. Further, in many ship tests the contact terminates when the relative speed between ice and the ship is zero—this means that the indentation speed varies between the ship speed and zero.

Overall, it is not clear if the failure mechanism in compressive tests and in indentation tests is the same. In compressive tests, the test specimen fails at a crack originating from small cracks present on the specimen surface or inside the specimen. The indentation ice failure is, on the other hand, a process where the failure itself is influenced by the process.

The introduction of tactile sensors has made the measurement of ice pressure possible on the whole nominal contact area. Much knowledge has been gained from several test series carried out using these tactile sensors. The development of the high-pressure area has received attention but less so the pressure on other areas or pressure in the ductile ice failure regime. This state of knowledge is reflected in the models developed for the ice pressure. The most common model—pressure–area relationship—is empirical. The effect of the failure mode on this relationship is not clear. It would be very interesting if laboratory or other controlled tests were carried out to improve the modelling of the ice pressure.

In most of the indentation tests the force–time history included a triangular pattern. This was, however, missing in some of the level sea-ice indentation tests in Hokkaido. The reason for the pattern is usually placed on brittle ice failure even if the test where this pattern was missing ice failure was described as brittle flaking. The observation of flaking is not very new; see, for example, Matlock *et al.* [49] or the indentation tests carried out at Cold Regions Research and Engineering Laboratory (CRREL) in the USA (for example, [50]). Indentation rate influences the failure so that at low indentation rates the ice failure is ductile (involving creep) and at higher rates brittle. Ice temperature influences the failure mode and thus also the shape of the force–time history. Indentation tests with quite warm sea ice suggest that the failure mode change occurs between rates 0.3 and 3 mm s^−1^. This would mean that practically all ship–ice interaction occurs in the brittle range. The triangular, pulsing force–time history causes structures to vibrate and is also, for this reason, an important research topic.

## Data accessibility

This article does not contain any additional data.
